# Wild Alaskan salmon supplementation increases 25-OHD levels in sled dogs

**DOI:** 10.30574/gscarr.2024.18.2.0036

**Published:** 2024-02

**Authors:** Kali Ann Striker, Scott Painter Jerome, Mary Ann Lila, Mari Høe-Raitto, Laura Kay Falkenstein, Lawrence Kevin Duffy, Kriya Lee Dunlap

**Affiliations:** 1 Department of Chemistry and Biochemistry, University of Alaska Fairbanks 900 Yukon Drive Fairbanks, AK 99775 USA.; 2 Institute of Arctic Biology, University of Alaska Fairbanks, 2140 Koyukuk Drive Fairbanks, AK 99775 USA.; 3 Plants for Human Health Institute, North Carolina State University, 600 Laureate Way Kannapolis, NC 28081.; 4 Alaska Gateway School District, PO Box 226, 1313.5 Alaska Highway Tok, AK 99780 USA.

**Keywords:** Canine athlete, Vitamin D, Parathyroid hormone, Vitamin D binding protein, Circumpolar North

## Abstract

Vitamin D deficiency affects canines and humans alike. Athletes are a particularly high-risk group. Further research regarding optimal intake and supplementation is needed to establish the parameters of vitamin D status in both humans and canines and to create a physiologically-relevant definition for vitamin D sufficiency. The objectives of this study were to (1) compare 25-OHD (25-hydroxy vitamin D) levels in sled dogs before and after receiving a diet supplemented with wild Alaskan salmon and (2) correlate biomarkers associated with vitamin D metabolism to 25-OHD levels in sled dogs. Plasma samples from 14 working sled dogs between 10 months and 7 years of age were collected before and after a 4-week supplementation with ≈0.45 kg of salmon/day. Samples were analyzed using enzyme-linked immunosorbent assays (ELISA) for parathyroid hormone and vitamin D binding protein (DBP); 25-OHD levels were measured via radioimmunoassay. 25-OHD and DBP in sled dogs significantly increased after a 4-week supplementation with salmon (P=0.0011 and 0.0367, respectively). Additional variations in 25-OHD were observed when separated by sex (P=0.0404) and age (P<0.0001). 57% of the dogs were deficient prior to the salmon supplementation and 14% at the completion of the study. Wild Alaskan salmon is one of the few food sources that provide appreciable amounts of vitamin D. A 4-week salmon supplementation in sled dogs was sufficient to improve 25-OHD concentrations in most sled dogs in this study. Confounding factors such as age and sex affect circulating 25-OHD levels.

## 1. Introduction

It is estimated that the prevalence of vitamin D deficiency (VDD) in people living above 37°N is around 39% but can be as high as 64.5% in populations living in higher latitudes [1]. This puts peoples of the circumpolar north at particularly high risk. Vitamin D deficiency in dogs is linked to many of the same health problems as humans, including several cancers, cardiovascular disease, kidney disease, impaired immune function, and cognitive decline. Young et al. (2017) estimated that 71% of all dogs are deficient [2]. Limitations to current knowledge of vitamin D in canines include the unestablished relationship between vitamin D intake and resulting physiological effects, the efficacy of supplementation methods, and dietary requirements [3]. The focus of this research was to determine the effects of a natural source of vitamin D, wild Alaska salmon, on vitamin D status in canine athletes.

Sufficient serum 25-hydroxyvitamin D (25-OHD) levels in dogs is thought to be above 100 ng/mL based on parathyroid hormone levels [4]. The only study that has assessed 25-OHD levels in sled dogs indicates that they were all deficient. Mean serum 25-OHD in a consecutive multi-day, long-distance sled dog race ranged from 57 to 87 ng/mL [5]. Following the requirements established by the Association of American Feed Control Officials (AAFCO), commercial dog foods must provide 500–5000 IU/kg of daily vitamin D, however many companies fail to account for the endogenous vitamin D in the ingredients being used. This issue, compounded with such a large recommendation range, has caused cases on both extremes— over-supplementation leading to toxicity and under-supplementation leading to developmental abnormalities, i.e. rickets. A study using a dosage of five times the daily recommended allowance (within safe upper limit) only yielded significant results after 9–10 weeks of supplementation, and still found apparently healthy dogs with insufficient plasma vitamin D levels [2].

Salmon is a staple in traditional Alaskan subsistence diets and is one of the few foods that contain appreciable amounts of vitamin D_3_ [1,6]. Wild Alaskan salmon has 500–1000 IU of vitamin D/100g. In comparison, farm-raised salmon only contains 100–250 IU of vitamin D/100g [7]. Geographic isolation in Alaska has fostered the rare preservation of traditional lifestyles; few populations exist today that still rely so heavily on subsistence foods and have historically low prevalence of diet-related diseases[8]. Alaskan villagers eat on average 4.8 kg of subsistence foods per week, 60% of which are finfish such as salmon [9]. Like their human counterparts, sled dogs living in the circumpolar north meet much of their energy demands from finfish, particularly salmon and white fish. Also like their Indigenous human counterparts, they rely on diet to meet their vitamin D requirements [4,10]. Dogs are thought to have limited ability to synthesize vitamin D in the skin, hypothesized to be due to an evolutionary adaptation to meat-based, vitamin D rich foods [10]. Industrialization in the 1900s that resulted in large-scale vitamin D deficiency in humans also negatively impacted vitamin D consumption in pet dogs corresponding to a shift to commercial foods. An analysis of serum 25-OHD in dogs fed 42 different commercial and homemade diets indicated that typical canine diets do not provide adequate amounts to meet sufficiency. Similarly, there is an inverse relationship with age and serum 25-OHD levels.

Although supplementation with vitamin D has been explored in dogs, only few studies are successful in raising 25-OHD in serum; this may be related to the bioavailability of vitamin D in the diet[2]. One study using fish oil, salmon oil, and a fortified dog biscuit only saw an increase in serum 25-OHD with the salmon oil supplement [11]. Vitamin D circulation is dependent on the vitamin D binding protein (DBP), which has been shown to have higher affinity for D_3_ compared to D_2_ [12]. Fish liver has an abundance of vitamin D stores, making fatty fish an excellent source of vitamin D_3_. In this study, using wild Alaskan salmon caught by gill net on the Yukon River we observed changes in plasma after 4 weeks of supplementation. To explore the relationship between vitamin D intake and resulting physiological status, we measure plasma parathyroid hormone (PTH) and vitamin D binding protein (DBP) in conjunction with 25-OHD after a 4-week salmon supplementation. PTH is responsible for the regulation of vitamin D metabolites, and the DBP is responsible for the transportation of metabolites among other physiological functions. Previous canine research has established the inverse relationship between vitamin D metabolites and serum PTH [13], however a DBP link has yet to be established in canines. The DBP has been acknowledged as an important factor in human health to regulate immune function and inflammation, as well as being used as a marker for chronic disease in canines [14]. The point at which decreasing PTH hits a plateau and an inverse relationship with 25-OHD begins has been suggested to represent a physiologically relevant point where vitamin D deficiency becomes sufficient. Clinical determination of vitamin D status should be based on this range with the PTH plateau [15].

## 2. Material and methods

### 2.1. Animals

Sled dogs (n=14) from a private mushing kennel in Salcha, Alaska (64N,146W) were used for this study. This study was approved by the University of Alaska’s Institutional Animal Care and Use Committee (IACUC, approval #1343101–5). All dogs underwent a similar training routine and were from the same genetic lineage. The dog owner/kennel manager is a dog musher, competing at the national and world levels for more than 3 decades. Maintaining an ideal body condition score is an important factor in performance. The kennel owner fed the dogs to maintain ideal body condition scores between 4–5 based on previous literature[16]. Body condition scores were checked before and after supplementation. All dogs acted as their own control throughout the study. Dogs were selected to provide diversity in both age and sex; there were 8 females and 6 males with ages ranging between 10 months old to 7 years old.

Our main analysis was pre and post supplementation. However, further analysis was conducted on the confounding factors of age and sex. Age was separated into 3 categories to observe variations based on developmental stages. Puppies were all 10 months old (n=4), adults were between ages 2–4 years old (n=7), and old dogs were 7 years or older (n=3).

### 2.2. Supplementation

Prior to treatment, sled dogs were fed with a high protein, high fat (32%,20%) commercial diet for performance canines with supplementation of corn oil and egg powder. This commercial diet is reported to have 2,221.12 IU/kg of Vitamin D in dry matter. An average size sled dog from this kennel (~50lbs) eats approximately 600g daily of dog food as fed, supplying approximately 1,333 IU of vitamin D daily before supplementation. After an initial baseline blood collection, the dogs’ diet was then supplemented with wild Alaskan chum salmon caught by gill net from the Yukon River. Approximately 0.45 kg of salmon was fed to each dog, replacing a large portion of the commercial food. The addition of salmon accounted for more than half of the total diet and dogs that were larger (eating more) received more salmon, but it remained proportionally equivalent between dogs. Additional vitamin D levels from wild Alaskan salmon likely provided between 2,250–4,500 IU/day based on reported levels that range between 500–1,000 IU in 0.1 kg [7]. This means during treatment dogs were receiving approximately 2,850–5100 IU/day of vitamin D. After 4 weeks of salmon supplementation a final blood collection was done.

### 2.3. Blood Collection

Eight mL of Blood was drawn by venipuncture before and after supplementation with a 21-gauge butterfly needle from the cephalic vein into a 10-ml syringe, and then transferred into a vacutainer containing the anticoagulant, EDTA. Within 30 minutes the samples were centrifuged at 3600RPM for 15 minutes, then the plasma layer was aliquoted and divided into 8 freezer vials per dogs to be stored at −80C for later analysis.

### 2.4. Biochemical Analysis

Canine specific enzyme-linked immunosorbent assays (ELISA) were used to quantify concentrations of PTH (MyBioSource), and DBP (Blue Gene) in plasma samples. The manufacturers’ instructions for lab protocol were followed. Absorbance was measured at 450nm with a microplate reader (BioTek Synergy HT). Sample concentrations were interpolated from standard curves determined with known concentrations of the respective proteins that were measured.

One sample from each dog was sent to Michigan State University Diagnostic Center for Population & Animal Health for 25-OHD analysis by radioimmunoassay (RIA). In 2020 a study associating vitamin D metabolites with DBP and proteinuria in dogs used both ELISA for DBP and RIA for serum 25-OHD, consistent with our study(17) and validated by literature(18). The same study also used multivariable linear regression models to determine associations with various clinical variables.

### 2.5. Data & Statistical Analysis

All samples were analyzed using GraphPad Prism statistical software (version 9.0.0) to evaluate differences between pre- and post-salmon supplementation. Differences in serum 25-OHD, PTH, and DPB concentrations between groups were evaluated by a paired t-test assuming Gaussian normal distribution, also consistent with literature [19,20]. Two-way ANOVA with Sidak’s multiple comparison was used to test variations based on sex and age independently due to treatment. Regression analyses were used for determining correlations between vitamin D levels with concentrations of PTH and DBP. Pearson r correlation was evaluated using 25-OHD concentrations as the independent factor, with the PTH and DBP as the dependent factors. Significance was determined using a p-value of <0.05.

## 3. Results

### 3.1. Plasma 25-OHD Concentrations

**Figure 1:**
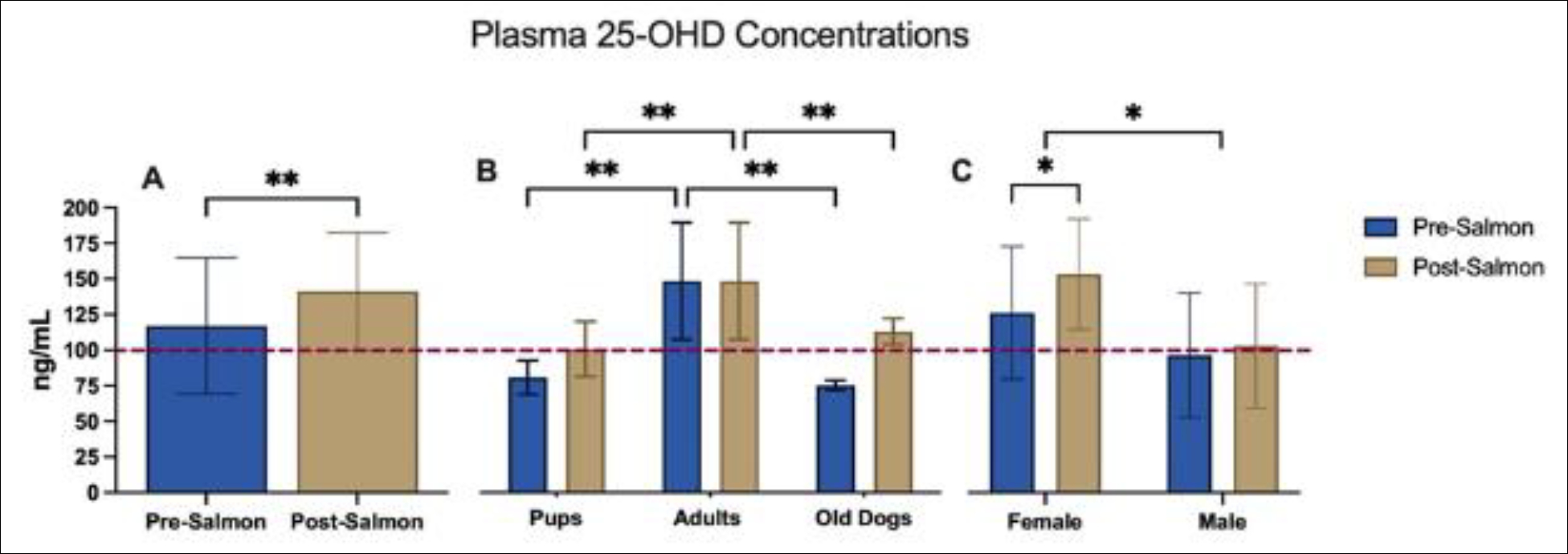
Plasma 25-OHD concentrations (±SD) in sled dogs before and after a 4-week treatment with salmon. Dashed line indicates the level suggested to be sufficient in dogs (100ng/ml). Graph A shows a significant increase in 25-OHD concentration before and after salmon supplementation (P=0.0011). Graph B indicates significant differences in 25-OHD concentrations according to age. There was a significant difference between puppies and adults for both pre and post treatment (P=0.002 and P=0.0001 respectively). Pups were 10 months of age. Old dogs were over the age of 7. Graph C shows a significant difference in 25-OHD between sexes (P=0.0403)

There was a significant increase in serum vitamin D concentrations after treatment with salmon (P=0.0011) (Figure 1A). The two-way ANOVA revealed significance in variation based on age (P≤0.0001); puppies were significantly different than in both pre- and post- treatment groups (P=0.0020 and 0.0001 respectively) (Figure 1B). Separation by sex yielded a significance in variation based on sex (P=0.0403) (Figure 1C).

### 3.2. Biomarkers

**Figure 2:**
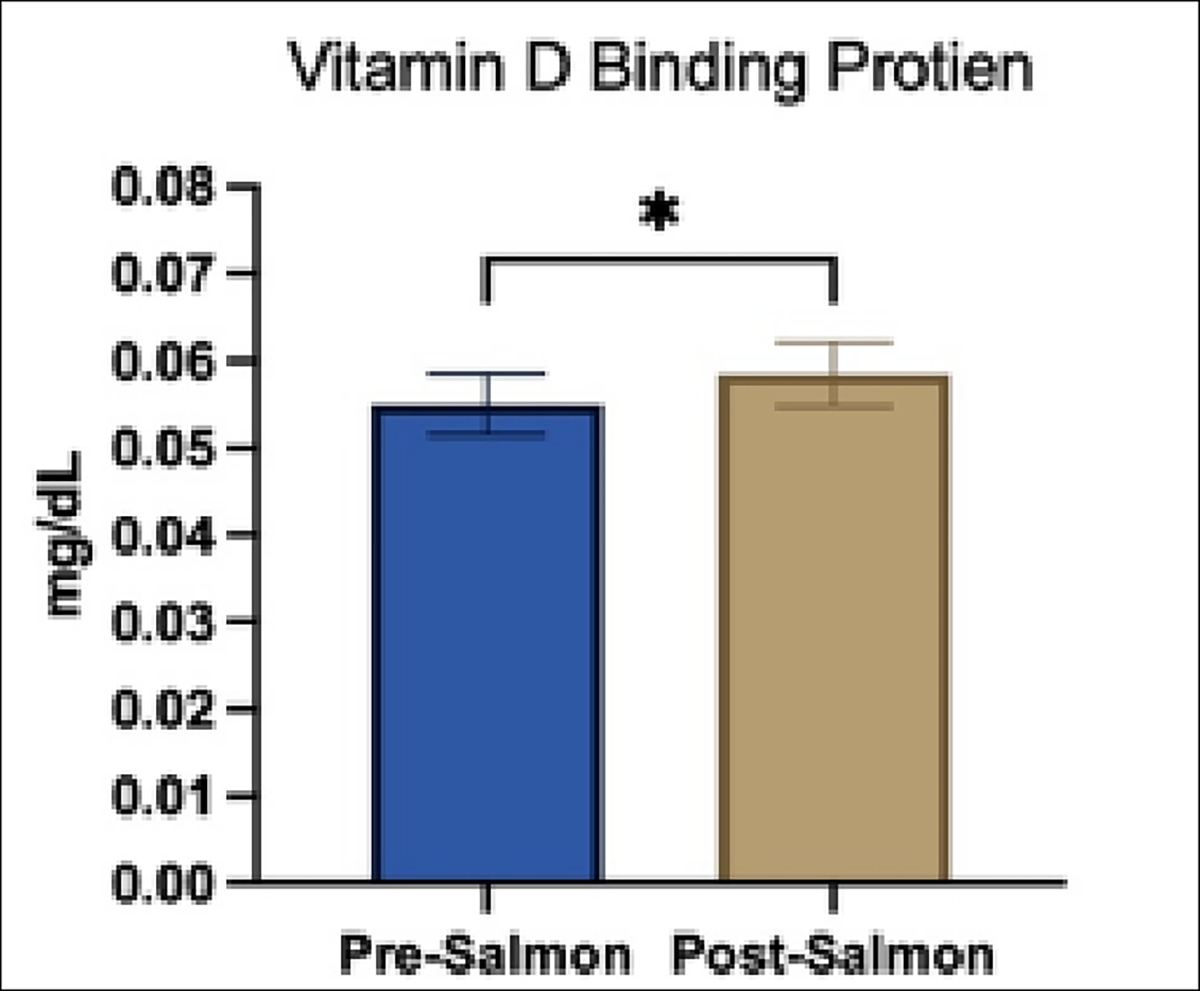
Plasma vitamin D binding protein (VDBP) concentrations (±SD) before and after a 4-week supplementation with salmon. There was a significant increase in VDBP concentration after supplementation(P=0.036)

There was no significant difference between parathyroid hormone levels before and after treatment. However, significance was found in DBP concentrations before and after treatment with salmon (P=0.0367) with no significance in correlation coefficient (Figure 2, Table 1).

### 3.3. Correlation of 25-OHD Concentration with Biomarkers

Analysis using the Pearson r correlation revealed no significant correlations for PTH or VDBP to concentrations of serum 25-OHD (Table 1).

## 4. Discussion

Supplementation using wild Alaskan salmon as a dietary source of vitamin D provided significant increases in serum 25-OHD concentration in sled dogs; furthermore, separation of dogs by confounding factors, like age and sex, yielded significant differences (Figure 1). These results highlight the importance of sex and developmental state on vitamin D status in canines. Although we did not see significant changes in PTH hormone concentrations, there was a significant increase in DBP concentrations after salmon supplementation (Figure 2).

When vitamin D status is low, the parathyroid hormone is triggered; this creates an inverse relationship between the vitamin D concentration and the PTH concentration. When vitamin D is sufficient, the PTH levels reach a plateau. The PTH plateau seen starting at 100ng/mL for 25-OHD has been reported in the literature and is suggested to be used as a more physiologically relevant definition of healthy vitamin D status in dogs [11]. A larger sample size or a larger distribution in vitamin D levels within this study population, i.e. to include more dogs with sufficient levels, may be needed to see this correlation. A similar correlation between 25-OHD and PTH is found in humans, however it occurs at a lower level of serum 25-OHD [21]. Therefore, dogs have a much higher vitamin D requirement per kg. Optimal health for vitamin D in dogs is seen at sufficient levels ranging between 100–120 ng/mL, anything less than 30 ng/mL is suggested to be VDD in dogs; these standards are set by laboratories like the Michigan Diagnostic Center for Population and Animal Health (MDCPAH). Before supplementation 57% of the dogs in this study were deficient compared to 14% after the addition of daily salmon to their diet. Our results are in line with the other study that analyzed vitamin D levels of sled dogs, which found that overall sled dogs are insufficient or deficient in vitamin D levels before and during an 8-day race; values ranged between 57–87 ng/mL throughout the race [19]. Athletes are suggested to be at increased risk for developing VDD, therefore sled dogs may have higher vitamin D needs compared to pet dogs [22].

Correlations between vitamin D status and DBP levels have not previously been observed in dogs [20]. This relationship should be further established to determine the physiological relevance of DBP levels. It has been shown that there are differences in bioavailability of vitamin D due to the source, i.e. diet or skin synthesis. The increase in vitamin D due to salmon specifically could be a result of the affinity of the DBP for the D_3_ from fish lipids. It has been reported that binding efficacy for D_3_ over D_2_ is preferred for the DBP [23].

Like humans, there is an inverse relationship with age and serum 25-OHD levels in dogs. Also, akin to humans, suggested dietary intake and serum sufficiency levels are not well-defined [3]. Of the 14 dogs in this study, only 3 were over the age of 7 but they exhibited significantly lower serum 25-OHD levels compared to younger adult dogs before supplementation. Post-supplementation, however, all 3 old dogs had reached sufficiency. Interestingly, the only dogs that did not reach sufficiency after salmon supplementation were 10-month-old pups. Sharp et al. (2015) evaluated the effects of several supplements on serum 25-OHD levels in dogs and reported that salmon oil significantly increased serum vitamin D concentration but that other fish oil supplementations did not have an effect[3]. Our results support the use of salmon as efficient source of vitamin D in dogs. Our results also highlight varying needs depending on developmental stages and likely, activity level.

## 5. Conclusion

The results of this study indicate that sled dogs are at high risk for vitamin D deficiency and insufficiency. Like their human counterparts, older animals are more at risk. Interestingly, this study revealed that pups, under the age of 1 year were also at higher risk for deficiency. While salmon supplementation improved vitamin D status in adult dogs, it did not bring vitamin D status to sufficiency in these young dogs. This indicates that vitamin D requirements may be higher in young, growing canines. Furthermore, previous research suggests that not all sources of vitamin D improve vitamin D status. This study shows that salmon supplementation can improve vitamin D status in dogs and should be considered as a treatment in dogs with vitamin D deficiency.

## Figures and Tables

**Table 1:** Summary of means and SEM before and after treatment with wild AK salmon. P-values reported from paired t-tests on each compound, asterisk represents significance with 95% confidence. Correlation coefficients are represented by (r) with significance at >.8. Concentrations are from plasma samples and units are reported

Compound(unit)	Pre-	Post-	P-value	SEM pre-	SEM post-	( r )
[25-OHD](ng/mL)	113.43	137.06	0.0011	12.362	10.687	0.8840
[PTH](pg/mL)	0.042	0.0420	0.9424	0.00030	0.00031	−0.2669
[DBP](mg/dL)	0.05503571	0.0585	0.0367	0.00094	0.00098	−0.1977
